# A Novel Colonial Ciliate *Zoothamnium ignavum* sp. nov. (Ciliophora, Oligohymenophorea) and Its Ectosymbiont *Candidatus* Navis piranensis gen. nov., sp. nov. from Shallow-Water Wood Falls

**DOI:** 10.1371/journal.pone.0162834

**Published:** 2016-09-28

**Authors:** Lukas Schuster, Monika Bright

**Affiliations:** University of Vienna, Department of Limnology and Bio-Oceanography, Althanstraße 14, A-1090 Vienna, Austria; National Cancer Institute, UNITED STATES

## Abstract

Symbioses between ciliate hosts and prokaryote or unicellular eukaryote symbionts are widespread. Here, we report on a novel ciliate species within the genus *Zoothamnium* Bory de St. Vincent, 1824, isolated from shallow-water sunken wood in the North Adriatic Sea (Mediterranean Sea), proposed as *Zoothamnium ignavum* sp. nov. We found this ciliate species to be associated with a novel genus of bacteria, here proposed as “*Candidatus* Navis piranensis” gen. nov., sp. nov. The descriptions of host and symbiont species are based on morphological and ultrastructural studies, the SSU rRNA sequences, and *in situ* hybridization with symbiont-specific probes. The host is characterized by alternate microzooids on alternate branches arising from a long, common stalk with an adhesive disc. Three different types of zooids are present: microzooids with a bulgy oral side, roundish to ellipsoid macrozooids, and terminal zooids ellipsoid when dividing or bulgy when undividing. The oral ciliature of the microzooids runs 1¼ turns in a clockwise direction around the peristomial disc when viewed from inside the cell and runs into the infundibulum, where it makes another ¾ turn. The ciliature consists of a paroral membrane (haplokinety), three adoral membranelles (polykineties), and one stomatogenic kinety (germinal kinety). One circular row of barren kinetosomes is present aborally (trochal band). Phylogenetic analyses placed *Z*. *ignavum* sp. nov. within the clade II of the polyphyletic family Zoothamniidae (Oligohymenophorea). The ectosymbiont was found to occur in two different morphotypes, as rods with pointed ends and coccoid rods. It forms a monophyletic group with two uncultured *Gammaproteobacteria* within an unclassified group of *Gammaproteobacteria*, and is only distantly related to the ectosymbiont of the closely related peritrich *Z*. *niveum* (Hemprich and Ehrenberg, 1831) Ehrenberg, 1838.

## Introduction

Ciliates exhibit a broad spectrum of symbiotic associations with unicellular eukaryotes and prokaryotes. The green microalga *Chlorella* (Trebouxiophyceae) is the most common genus of unicellular algae that lives in symbiosis with freshwater protists, such as ciliates and amoebae [1,2]. Apart from algal eukaryotes, few other, heterotroph eukaryotic endosymbionts are known, including representatives of Microsporidia and trypanosomatid parasites (Kinetoplastea) infecting the cytoplasm or the macronucleus [3,4]. Prokaryotic symbionts with phototrophic, chemoautotrophic and heterotrophic metabolism in ciliates (e.g. [5–15]) comprise a variety of *Proteobacteria* and some archaea [16]. The symbionts are found both as ectosymbionts covering the surface of the ciliates or as endosymbionts colonizing almost all compartments of the ciliated cell [3,17–19].

Chemosynthetic symbioses are ubiquitous in marine environments, ranging from hydrothermal vents, whale and wood falls, cold seeps, mud volcanoes, and continental margins, to shallow-water coastal sediments [20–22]. The presence of an inverse gradient of reduced energy sources, such as sulfide and methane, in close proximity to oxidants, such as oxygen, nitrate, and sulfate, is a common denominator to all these environments [22]. It is at the oxic-anoxic interface where chemosynthetic symbioses prosper [20,23,24]. A variety of animal hosts from several different phyla with chemosynthetic symbionts are known [22]. Among ciliates, *Kentrophoros* Sauerbrey, 1928 (Karyorelictea) [25–30], *Folliculinopsis* Faure-Fremiet, 1936 (Heterotrichea) [31], and *Zoothamnium* Bory de St. Vincent, 1824 (Oligohymenophorea) [32–37] were found being associated with chemosynthetic bacteria.

Representatives of *Zoothamnium* are generally found in aquatic environments from freshwater to seawater. They are sessile and attached to seagrass, macroalgae, animals, or various substrates such as stones or wood [38]. Most representatives of this genus live a solitary life, but some species have been described with epibiotic organisms covering the host [39–50].

In the first classification of Ehrenberg [37], the genus *Zoothamnium* was placed within the family Vorticellidae Ehrenberg, 1838. In 1951, Sommer [51] created the family Zoothamniidae, with the defining character of a continuous spasmoneme. Corliss [52] did not recognize the Zoothamniidae and placed *Zoothamnium* within the Vorticellidae again. Lynn and Small [53] recognized *Zoothamnium* as the only genus within the Zoothamniidae, based on the following morphological characteristics: colonial habit, continuous spasmonemes, and entire colony contractile. In 2008, Lynn [38] added seven additional genera to the family Zoothamniidae (i.e. *Craspedomyoschiston* Precht, 1935; *Haplocaulus* Warren, 1988; *Mesothamnium* Jankowski, 1985; *Myoschiston* Jankowski, 1985; *Pseudohaplocaulus* Warren, 1988; *Zoothamnioides* Schoedel, 2006; and *Zoothamnopsis* Song, 1997). The distinguishing morphological characters were the presence of a stalk that contracts in a ‘zig-zag’ pattern due to a spasmoneme that runs uninterrupted throughout the entire colony [38] rather than a helical pattern as in the Vorticellidae.

On a molecular basis, however, *Zoothamnium* exhibits a high genetic diversity. Phylogenetic analyses of the 18S rRNA gene sequences, the ITS1-5.8S-ITS2 region sequences and the combined 18S + ITS region sequences showed clearly that the genus *Zoothamnium* is polyphyletic [54–61]. Furthermore, the monophyly of *Zoothamnium* was clearly rejected by performing approximately unbiased tests [61]. Therefore, in the current classification, *Zoothamnium* is divided into five clades (see [61]).

During an investigation of *Zoothamnium niveum* (Hemprich and Ehrenberg, 1831) Ehrenberg, 1838 and its sulfide-oxidizing ectosymbiont “*Ca*. Thiobios zoothamnicoli” Rinke *et al*., 2006 [32–37] from shallow-water wood falls in the Northern Adriatic Sea, we isolated a further *Zoothamnium* species. Bacteria, indicative of a symbiotic association, covered the entire colony, except for the most proximal part. Here, we describe this new ciliate symbiosis. Morphological and phylogenetic analyses suggested that the ciliate represented a novel species of *Zoothamnium* and the bacteria covering the ciliate represented a novel bacterial genus.

## Materials and Methods

### Ethics statement

No specific permissions were required for the listed locations as they are publicly accessible. Furthermore, we confirm that our field studies did not involve endangered or protected species.

### Sample collection and fixation

**Fig 1:**
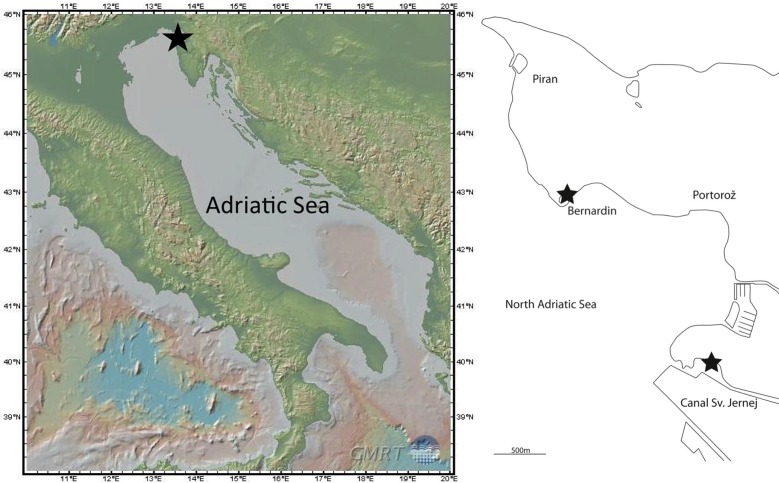
Sampling sites in the North Adriatic Sea. Map of the Adriatic Sea with details of the sampling sites (star) at the Bernardin harbor (star) and the canal Sv. Jernej (star), Slovenia. The map was created using the GMRT MapTool (www.marine-geo.org/tools/GMRTMapTool [62]).

**Fig 2:**
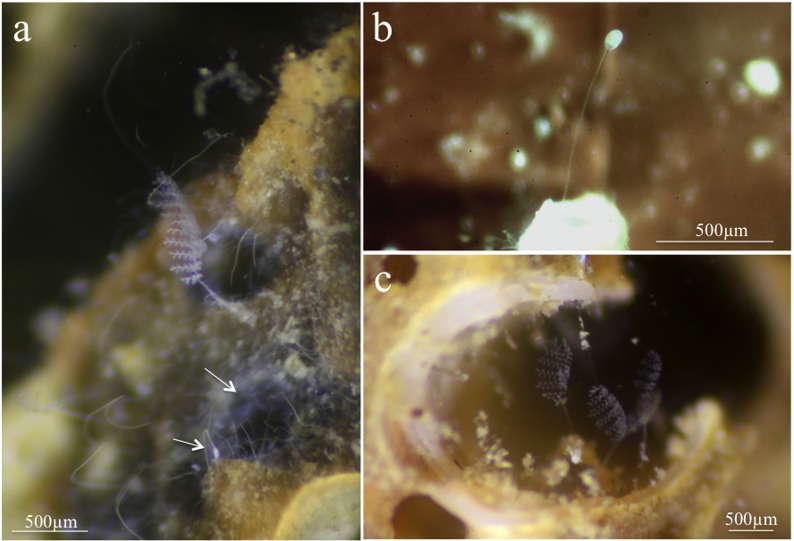
*Zoothamnium ignavum* sp. nov. colonies on sunken wood. a, c) *Z*. *ignavum* sp. nov. colonies, b) top terminal zooid; white arrows denote free-living bacteria colonizing the same wood surfaces as *Z*. *ignavum* sp. nov.

Colonies of *Zoothamnium ignavum* sp. nov. were collected from sunken wood at a depth between 1 and 1.5 m in the North Adriatic Sea (Mediterranean Sea) at two locations in the vicinity of Piran, Slovenia, the Bernardin harbor in 2014 and the canal Sv. Jernej in 2014 (Figs 1 and 2). Colonies were frozen in liquid nitrogen and stored at– 80°C for DNA extraction, or fixed and stored in 100% ethanol for DNA extraction and FISH, respectively, or fixed and preserved in a modified Trump’s fixative (2.5% glutaraldehyde, 2% paraformaldehyde in sodium cacodylate 0.1 mol L^-1^; 1100 mOsm L^-1^; pH 7.2) for up to six months until further treatment for scanning electron microscopy.

### Microscopic studies

Freshly collected colonies were studied with bright-field and differential interference contrast optics on a Leica DM2000 microscope. Measurements were taken from living colonies and individual cells. In order to reveal the kinetosomes and nuclei, the pyridinated silver carbonate impregnation technique after Fernández-Galiano [63] was used. Bacteria were stained using the LIVE/DEAD® *Bac*Light™ Bacterial Viability Kit (Thermo Fisher Scientific). Drawings were made from photographs taken with the Leica DM2000 microscope equipped with a Leica DFC295 camera. Photographs of living colonies on wood were made with a Canon EOS 550D camera on a BMS 144 stereomicroscope.

### DNA extraction, polymerase chain reaction (PCR) and sequencing

DNA was extracted from 13 individual colonies using the KAPA Express Extract Kit (KAPA Biosystems), with slight modifications of the reaction volume: the total volume was 20 μL, consisting of 2 μL Express Extract Buffer, 0.4 μL Express Extract Enzyme, and 17.6 μL dH_2_O. Lysis incubation was done at 75°C for 20 min, followed by an enzyme inactivation step at 95°C for 5 min. The 16S rRNA genes were amplified by PCR using the universal bacterial primers 27 forward and 1492 reverse [64]. The 18S rRNA genes were amplified using the universal eukaryotic primers 82 forward [65] and Medlin B reverse [66].

The obtained PCR products from each colony were cloned separately using the TOPO-TA cloning kit (Invitrogen) according to the manufacturer’s instructions. For screening of the 16S rRNA and 18S rRNA genes, 10 to 15 clones each were picked and controlled for the correct size by PCR with the M13 forward and M13 reverse primers (Invitrogen). Polymerase chain reaction products of the correct size for the 16S rRNA gene (~1,500 nt) and for the 18S rRNA gene (~1,800 nt) were fully sequenced via Sanger sequencing and further analyzed using the program CodonCode Aligner (CodonCode Corporation; www.codoncode.com).

### Host and symbiont phylogenetic analyses

The obtained 16S and 18S rRNA gene sequences were compared with the National Center for Biotechnology information NCBI (http://www.ncbi.nlm.nih.gov) database using BLAST [67]. For phylogenetic analyses of 18S rRNA gene sequences, all sequences available for *Zoothamnium* spp. plus some other Peritrichia were included (66 sequences).

For phylogenetic analyses of 16S rRNA gene sequences, all BLASTn hits longer than 1400 nt with sequence identities higher than 90% to “*Ca*. Navis piranensis” gen. nov., sp. nov., including the sequences of species belonging to the “Thiobios Group” as defined in a previous study [39], and four sequences belonging to the *Thiotrichales* were included. As outgroup, four sequences of non *Gammaproteobacteria* were included. S1 Table provides accession numbers of sequences included in the phylogenetic analyses of 18S rRNA gene sequences. S2 Table provides accession numbers of sequences included in the phylogenetic analyses of 16S rRNA gene sequences. Sequences were aligned by MAFFT using the Q-INS-i strategy that considers the secondary structure of RNA [68] and alignments were checked manually. For phylogenetic analyses, we evaluated the optimal nucleotide substitution model based on the Akaike information criterion using MrModeltest2 [69]. The general time-reversible (GTR) model with invariable sites (I) and a Γ-correction for site-to-site rate variation was selected for all analyses. A 50% majority-rule Bayesian inference tree was constructed with MrBayes 3.2.6 [70]. The chain length was 10000000 generations with trees sampled every 100 generations. The first 2500 trees were discarded as burn-in. The maximum likelihood analyses were carried out using the packages phangorn version 2.0.4 [71] and ape version 3.5 [72] in R version 3.2.2 [73]. Node robustness was assessed by performing bootstrap in ML analyses and calculating posterior probabilities in Bayesian inferences. Bootstrap support and posterior probabilities of at least 70% are indicated at the nodes of the trees. Trees were rooted by the mid-point technique.

### 16S rRNA symbiont-specific probe design and fluorescence *in situ* hybridization

The 16S rRNA bacterial gene sequences were added to the SILVA database [74] and two specific probes were designed with the ARB software package [75]. Probe specificity was checked against the ARB database and the Ribosomal Database Project by the implemented tool Probe Match [76]. Both probes showed, at least, one mismatch to all other 16S rRNA sequences available in the public databases. The nucleotide sequences of the newly designed probes ZIS645 and ZIS832 are available at probeBase ([77]; www.microbial-ecology.net/probebase).

Colonies fixed and stored in 100% ethanol were embedded in LR-white resin (London Resin Co.) and semi-thin sections (1 μm thickness) were prepared using an Ultracut E, Reichert-Jung ultramicrotome. FISH probes were labeled on their 5’ end with the fluorescent dyes Cy3 or Cy5 (Table 1). Optimal hybridization conditions for the newly designed specific probes ZIS645 and ZIS832 were determined by applying a series of formamide concentrations (0 to 35%) in the hybridization buffer [78]. Positive and negative hybridization controls were the EUBMix probe set consisting of EUB338, EUB338II and EUB388III [79,80], targeting most Bacteria, and the probe Non338, complementary to EUB338 [81]. 4’,6-Diamidino-2-phenylindole (DAPI) was used as a counterstain. Microscopic analyses were performed with a Zeiss Axio Imager M2 epifluorescence microscope.

**Table 1:** Probes used for fluorescence *in situ* hybridization.

probe	specificity	sequence	reference
EUB338	most Bacteria (EUBI+II+III)	5’-GCT GCC TCC CGT AGG AGT-3’	[79]
EUB338II	most Bacteria (EUBI+II+III)	5’-GCA GCC ACC CGT AGG TGT-3’	[80]
EUB338III	most Bacteria (EUBI+II+III)	5’-GCT GCC ACC CGT AGG TGT-3’	[80]
NON338	negative control	5’-ACT CCT ACG GGA GGC AGC-3’	[81]
ZIS832	*Z*. *ignavum* ectosymbiont	5’- GCT TAT ATC GCT CCC AAC A-3’	this study
ZIS645	*Z*. *ignavum* ectosymbiont	5’- ACC AGA CTC TAG TCA GCC A-3’	this study

### Scanning electron microscopy (SEM)

Thirteen colonies were fixed in a modified Trump’s fixative and stored in the fixative up to six months. A graded series of acetone was used for dehydration for 10 min each, followed by 1:1 acetone/hexamethyldisilazane (HMDS) and 100% HMDS for 10 min each, followed by air-drying the colonies. Afterwards, colonies were mounted on stubs and sputter coated with gold/palladium. A JOEL IT300 (Germany) scanning electron microscope was used to view the colonies.

### Nomenclatural Acts

The electronic edition of this article conforms to the requirements of the amended International Code of Zoological Nomenclature, and hence the new names contained herein are available under that Code from the electronic edition of this article. This published work and the nomenclatural acts it contains have been registered in ZooBank, the online registration system for the ICZN. The ZooBank LSIDs (Life Science Identifiers) can be resolved and the associated information viewed through any standard web browser by appending the LSID to the prefix http://zoobank.org/. The LSID for this publication is: urn:lsid:zoobank.org:pub:3B25B012-1ABF-4767-B2D7-0A95C26E5DF9. The electronic edition of this work was published in a journal with an ISSN, and has been archived and is available from the following digital repositories: PubMed Central, LOCKSS.

## Results

### Zoothamnium ignavum sp. nov.

SYSTEMATICS

The ciliate classification follows Lynn [38].

**Table:** 

Phylum:	Ciliophora Doflein, 1901
Class:	Oligohymenophorea de Puytorac *et al*., 1974
Subclass:	most Bacteria (EUBI+II+III)
Order:	most Bacteria (EUBI+II+III)
Family:	Zoothamniidae Sommer, 1951
Genus:	*Zoothamnium* Bory de Saint-Vincent, 1824

#### Diagnosis

*Zoothamnium* species with alternately branched stalk; zooids alternate on branches; three different types of zooids: microzooids (“trophic stage”), macrozooids (“telotroch stage”), terminal zooids; microzooids bulgy, inverted bell-shaped; macrozooids roundish to ellipsoid, located only on the most proximal part of the branches; top terminal zooid on the tip of the stalk, terminal zooids of the branches on the proximal end of each branch; undividing terminal zooids similar to microzooids in shape, dividing terminal zooids ellipsoid; macronucleus of microzooids S-shaped, showing irregular thickness; macronucleus of macrozooids extended through the whole cell, band-like with constant diameter; macronucleus of dividing terminal zooids S-shaped, regular thickness, filling almost the entire cell; in each zooid one orally located contractile vacuole present; a telotrochal band of one circular row of barren kinetosomes present aborally.

#### Type locality

The holotype and 12 paratypes of *Zoothamnium ignavum* sp. nov. were collected in July 2015 from sunken, degrading wood found in about 1 m depth in the canal of Sv. Jernej (North Adriatic Sea; 45° 29’ 52.7” N, 13° 35’ 36.7” E; water temperature: 24.8–31.5°C, salinity: 35.9, pH: 8.3). Additional material was collected in May 2014 in the harbor of Bernardin at a depth of 1.5 m (North Adriatic Sea; 45° 30' 52.4" N, 13° 34' 21.8" E; water temperature: 17.2–20°C, salinity: 18.6, pH: 8.26).

#### Type specimens

The holotype #5613 and nine paratypes #5614 –#5622 fixed in a modified Trump’s fixative (2.5% glutaraldehyde, 2% paraformaldehyde in sodium cacodylate 0.1 mol L^-1^, 1100 mOsm L^-1^; pH 7.2) were embedded in glycerol and mounted on microscope slides. Additionally, 10 paratypes #20443 were fixed in absolute ethanol. The type material was deposited at the Naturhistorisches Museum, Wien (Austria).

#### Gene sequence

The sequence of the 18S rRNA eukaryote gene of *Zoothamnium ignavum* sp. nov. was deposited in the GenBank database under accession number KX669262.

#### Etymology

The Latin adjective *ignav*^.^*us*, *-a*, *-um* [m, f, n] refers to the contraction behavior of this species, as in comparison to its close relative *Zoothamnium niveum* it seems to be ‘idle’.

#### Description

**Fig 3:**
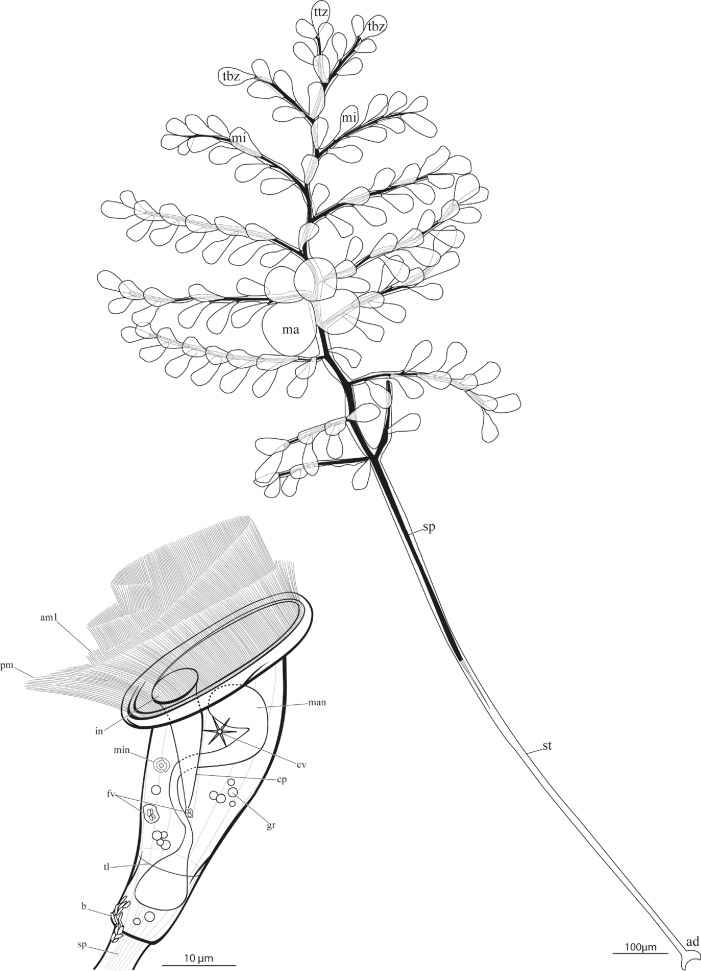
*Zoothamnium ignavum* sp. nov. Drawing of the colony and the microzooids in extended condition. mi: microzooid, ma: macrozooid, ttz: top terminal zooid, tbz: terminal branch zooid, sp: spasmoneme, st: stalk, ad: adhesive disc, pm: paroral membrane (haplokinety), am1: adoral membranelle 1 (polykinety), in: infundibulum, cp: cytopharynx, man: macronucleus, min: micronucleus, fv: food vacuole, cv: contractile vacuole, gr: granules, tl: telotrochal band, b: bacteria.

**Fig 4:**
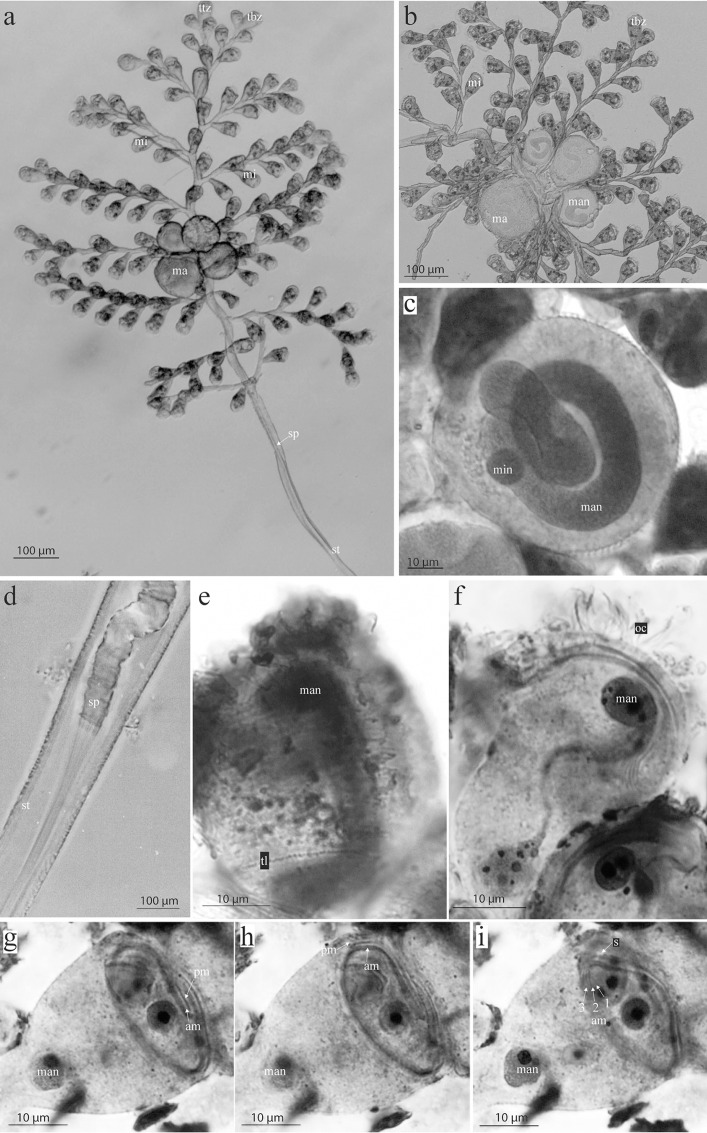
Micrographs of living and silver-stained *Zoothamnium ignavum* sp. nov. colonies. a, b) General view of living colonies, c) macrozooid, d) stalk with the end of the spasmoneme splitting up into bands, which bundle towards the proximal end of the colony, e) terminal branch zooid, f) microzooid, g-h) oral ciliature of the microzooids. a, b, d) living colonies. c, e-i) silver-stained colonies. ttz: top terminal zooid, tbz: terminal branch zooid, mi: microzooid, ma: macrozooid, sp: spasmoneme, st: stalk, man: macronucleus, min: micronucleus, tl: telotrochal band, pm: paroral membrane (haplokinety), am1-3: adoral membranelles 1–3 (polykineties), s: stomatogenic kinety.

The colony is composed of three different types of zooids, which are connected by a common stalk: (i) microzooids (“trophic stage”), (ii) macrozooids (“telotroch stage”) and (iii) terminal zooids (Figs 3 and 4). The microzooids are located along the entire branch, the macrozooids are restricted to the most proximal parts of the branches. The terminal zooids are considered to be the only zooids capable of longitudinal fission. They are located on the distal end of the stalk (top terminal zooid) and produce the terminal zooids of each branch (terminal zooids of branches), which then produce the microzooids, and on the proximal part of the branches the macrozooids. Due to the divisions, the colony can grow up to 1.8 mm in length. The number of macrozooids within each colony is variable, although colonies were often found having three to four macrozooids on one branch.

The core of the stalk is a contractile spasmoneme that runs uninterrupted throughout the entire colony, through the stalk and branches into each individual cell (zooid). This spasmoneme allows a simultaneous contraction of the colony and the oral side of each zooid. The end of the spasmoneme within the stalk splits up into bands, which bundle towards the proximal end of the stalk. Only the most basal part of the stalk and the adhesive disc lack a spasmoneme. The contraction occurs in a typical zigzag pattern and takes place rapidly while the subsequent extension is much slower. Merely in the expanded condition of the colony, the cilia of the oral apparatus have been observed to beat. Furthermore, younger zooids on the distal end of the stalk have been observed to be more active than older ones at the proximal end.

The branches occur alternating on the stalk. The stalk diameter increases from about 15 μm at the top end of the colony to about 23 μm at the location of the first and oldest branches, and decreases to about 11 μm at the basal end of the colony. Located in the stalk, the spasmoneme diameter is 4.5 μm at the top end of the colony, it increases to 5.5 μm at the level of the first branch, and decreases to 4 μm, where it ends at around 70% of the total stalk length. At the end, it splits up into bands and bundles towards the most proximal end of the colony. Divided into stalk with branches and stalk without branches containing a spasmoneme and lacking a spasmoneme, the relative lengths are 40%, 30%, and 30% (S1 Fig). The youngest and shortest branches are found at the top end of the colony. Throughout the colony, the distance between the branches varies between 48 and 100 μm. The average diameter of the branches is about 9.2 μm, with the corresponding diameter of the spasmoneme of about 2.9 μm. On the branches, the microzooids occur alternating. The distance between microzooids varies from 18 to 34 μm.

**Fig 5:**
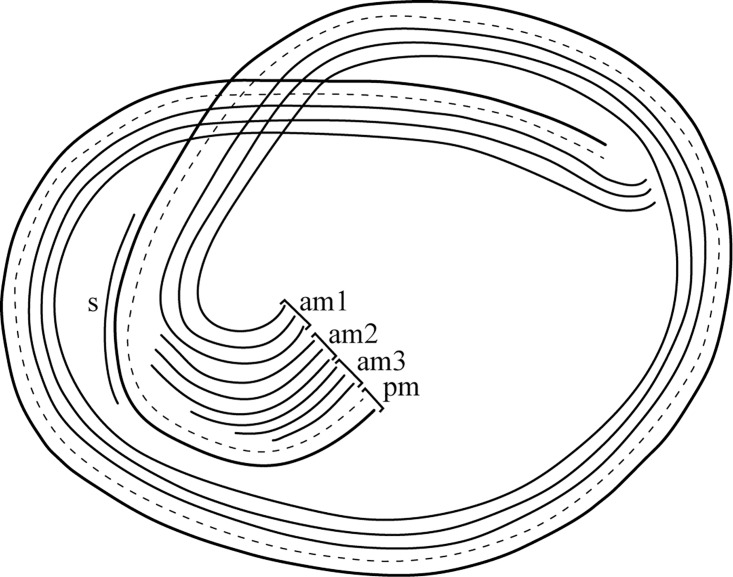
Schematic illustration of the oral ciliature of the microzooid of *Zoothamnium ignavum* sp. nov., viewed from the oral pole. pm: paroral membrane (haplokinety), am1-3: adoral membranelles 1–3 (polykineties), s: stomatogenic kinety.

Typically, the extended microzooids have a bulgy, bell-shaped form (average length 39.4 μm, SD 3 μm; average oral width 28.8 μm, SD 3.1 μm; average aboral width 8.2 μm, SD 1.9 μm; n = 20; Fig 3). At the oral side, the microzooids are strongly asymmetric. In the retracted stage, the peristome with the peristomial disc and the single oral lip are withdrawn giving the microzooid a more symmetrical appearance. The S-shaped macronucleus, having a variable number of constrictions, extends throughout the microzooid. A small, roundish micronucleus is found adjacent to the macronucleus. On the opposite side of the infundibulum, one contractile vacuole is present. The cytoplasm is packed with tiny, dense granules (average diameter 3 μm). The pellicula of the microzooids is plain, with a striped silver line system (width of the striae 0.2–0.4 μm). The oral ciliature consists of a paroral membrane (haplokinety), three adoral membranelles (am 1–3; polykineties), and one short stomatogenic kinety (germinal kinety). The paroral membrane lies outside the innermost adoral membranelle (am 1). Viewed from inside the cell, the paroral membrane and the adoral membranelle (am 1) run jointly 1¼ turns in a clockwise direction around the peristomial disc and run into the infundibulum, where they make another ¾ turn. There, a short stomatogenic kinety of barren kinetosomes is present outside the paroral membrane. The innermost adoral membranelle (am 1) extends to the posterior end of the infundibulum, where it is accompanied by two shorter adoral membranelles (am 2, am 3) (Figs 4 and 5). At around two-thirds distance from the peristomial disc, the somatic ciliature, consisting of a single irregular row of barren kinetosomes forming the telotrochal band, is found.

Similar to the microzooids, terminal zooids have a bulgy, bell-shaped form, resembling the microzooids in shape and morphological characteristics (average length 51.6 μm, SD 9.1 μm; average oral width 23.3 μm, SD 4.5 μm; average aboral width 11.3 μm, SD 3.2 μm; n = 20). However, some terminal zooids have a more ellipsoid shape (average core width 29.2 μm, SD 2.1; n = 4). These are thought to be in a dividing stage, having a very large macronucleus filling up almost the whole cell body.

The macrozooids are roundish to ellipsoid with a diameter between 35 and 86 μm. The macronucleus appears very thick and constant in diameter, filling up almost the whole cell. One micronucleus lies adjacent to it. Orally, a large contractile vacuole is present. A cytopharynx does not appear to be developed, although some cytopharyngeal microtubuli are present. The pellicula of the macrozooids has bands transverse to the oral-aboral axis of the cell. The width of the striae is correlated with the size of the cell and ranges from 0.9 to 2 μm. Aborally a telotrochal band with several circular rows of kinetosomes is present. It is found in the same position as the single circular row of kinetosomes in the microzooids. As long as the macrozooids remain attached to the colony, the telotrochal band is only partly ciliated. In all free-swimming macrozooids, the telotrochal kinetosomes are fully ciliated.

#### Remarks

*Zoothamnium ignavum* sp. nov. resembles *Z*. *alternans* Claparède and Lachmann, 1859 redescribed from a population from Qingdao, China [44] in the shape of the colony, the branching pattern, and the size of the microzooids (Table 2). However, several characters are conspicuously different between Z. *ignavum* sp. nov. and Z. *alternans* and clearly distinguish these two species (Table 2, S1 Fig). The shape of the macronucleus in the microzooids is S-shaped in *Z*. *ignavum* sp. nov. while it is J-shaped in *Z*. *alternans*. In *Z*. *alternans* the infundibular polykineties in the microzooids perform a full turn around the infundibulum and extend posteriorly to the end of the infundibulum [44]. In contrast, they are much shorter in *Z*. *ignavum* sp. nov. and perform only a ¾ turn around the infundibulum, similar to the infundibular polykineties in *Z*. *niveum* ([82], S1 Fig). Distinguishing three parts of the stalk from the top to the bottom in 1) stalk with branches and spasmoneme, 2) stalk with spasmoneme and without branches, and 3) stalk without spasmoneme and without branches, the relative lengths of *Z*. *ignavum* sp. nov. colonies are about 40%, 30%, and 30%. In contrast, in *Z*. *alternans* they are about 80%, 10%, 10%. Thus, the lower part of the stalk (from the adhesive disc to the lowest branch) is much shorter in *Z*. *alternans* (about 20% of the total stalk length) than in *Z*. *ignavum* sp. nov. (more than 50% of the total stalk length) (S1 Fig).

**Table 2:** Colony dimensions and characteristics of *Zoothamnium ignavum* sp. nov. in comparison to *Z*. *alternans* QD and *Z*. *niveum*. st + br + sp: stalk with branches and spasmoneme; st–br + sp: stalk without branches and with spasmoneme; st–br–sp: stalk without branches and spasmoneme.

		*Z*. *ignavum* sp. nov.	*Z*. *alternans* QD	*Z*. *niveum*
**colony**	size [mm]	1.8	1.2	15
	st + br + sp [%]	40	80	60
	st–br + sp [%]	30	10	35
	st–br–sp [%]	30	10	5
**microzooids**	length [μm]	39.4 ± 3	40–56	59.8 ± 6
	oral width [μm]	28.8 ± 3.1	26–32	18.7 ± 3
	aboral width [μm]	8.2 ± 1.9	-	7.4 ± 2.4
	oral ciliature	1 ½ turns around the peristomial disc, infundibular kineties perform a ¾ turn around infundibulum	1 ⅓ turns around the peristomial disc, infundibular kineties perfom 1 full turn around infundibulum	1 ¼ turns around the peristomial disc, infundibular kineties perform a ¾ turn around infundibulum
	shape of macronucleus	S-shaped	J-shaped	Horseshoe-shaped
**macrozooids**	diameter [μm]	35–86	70–90	20–150
	reference	this study	[44]	[36,82]

Besides *Z*. *ignavum* sp. nov. and *Z*. *alternans*, also *Z*. *niveum* and *Z*. *plumula* Kahl, 1932 (syn. *Z*. *plumosum* Perejaslawzewa, 1858) have an alternate branching pattern. However, in *Z*. *plumula* the microzooids are located regularly in pairs along the branches and macrozooids are completely absent [83–85].

In *Z*. *niveum*, the colony resembles a feather, which can reach a length of up to 1.5 cm, making it by far the largest representative of this genus. In addition, the microzooids of this species are slightly larger than those of *Z*. *ignavum* sp. nov., are slender in shape, and exhibit a pronounced asymmetric lobe (Table 2). Also, the relative lengths of the stalk are different (60%, 35%, and 5%) compared to the proportions of *Z*. *ignavum* sp. nov. colonies (Table 2, S1 Fig). Furthermore, *Z*. *niveum* is characterized by an obligate association with the thiotrophic, ectosymbiotic bacterium “*Ca*. Thiobios zoothamnicoli”. Due to the sulfur storage of these bacteria [33,35–37,86,87], the whole colony appears in a bright white color, making it easily distinguishable from other *Zoothamnium* species, including *Z*. *ignavum* sp. nov.

*Z*. *pelagicum* Du Plessis, 1891, in contrast, has no alternating but rather a pinnate pattern of branching and no adhesive disc. This species is a planktonic ciliate and therefore easily distinguishable from other *Zoothamnium* species, which are found attached to various substrates or other living organisms [55,88–90].

### The 18S rRNA eukaryote gene sequence and phylogenetic analyses

**Fig 6:**
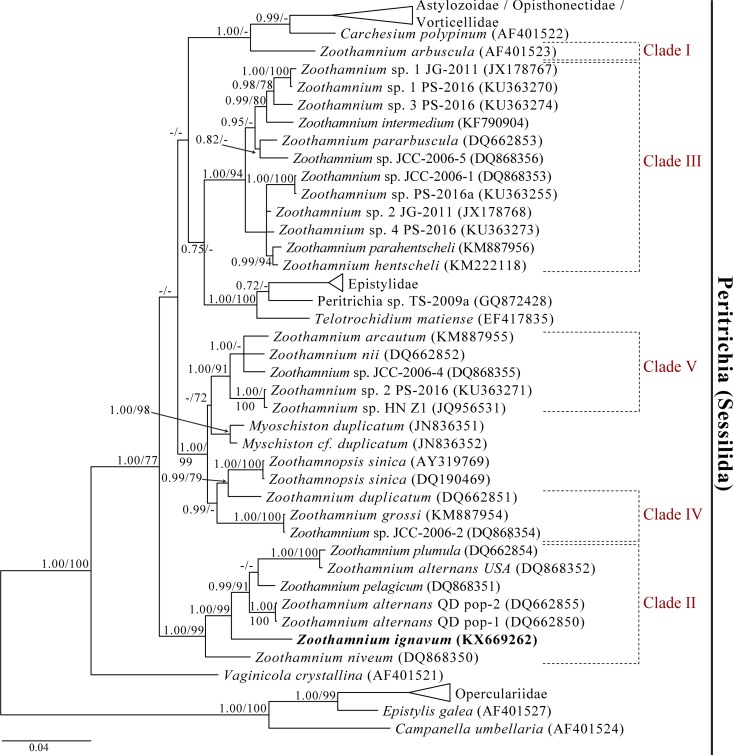
Bayesian inference tree inferred from the nucleotide sequences of the small subunit rRNA gene showing the phylogeny of *Zoothamnium ignavum* sp. nov. Numbers at nodes represent Bayesian posterior probabilities and ML bootstrap values. The scale bar corresponds to 4 substitutions per 100 nt positions.

The 18S rRNA gene sequences of the 13 *Z*. *ignavum* sp. nov. colonies examined shared over 99.6% sequence identity, indicating that they all belonged to the same species. The obtained sequences had a total length of 1,653 nt. In all phylogenetic analyses, the *Z*. *ignavum* sp. nov. sequence falls within the class Oligohymenophorea of the phylum Ciliophora and forms a monophyletic group (clade II) with *Z*. *alternans* populations from Qingdao (China) and USA, *Z*. *niveum*, *Z*. *pelagicum*, and *Z*. *plumula* (Fig 6). Based on the 18S rRNA gene sequence similarity, the closest relative is *Z*. *alternans* from Qingdao, with 96.7% sequence identity.

### “*Candidatus* Navis piranensis” gen. nov., sp. nov.

SYSTEMATICS

**Table:** 

Phylum:	*Proteobacteria* Stackebrandt *et al*., 1988
Class:	*Gammaproteobacteria* Garrity *et al*., 2005
Order:	Unclassified
Family:	Unclassified

#### Type locality

Same as for the host species *Zoothamnium ignavum* sp. nov.

#### Gene sequence

The sequence of the 16S rRNA bacterial gene of “*Candidatus* Navis piranensis” gen. nov., sp. nov. was deposited in the GenBank database under accession number KX669263.

#### Etymology

The Latin noun *nav*^.^*is*, *-is* [f] refers to the morphology of the symbiont, rod-shaped with pointed ends, similar to a boat. The species name refers to the location where the symbiosis was found (Piran, Slovenia) and was used as a Latin adjective *piranens*^.^*is*, *-is*, *-e* [m, f, n].

### The 16S rRNA bacterial gene sequence

**Fig 7:**
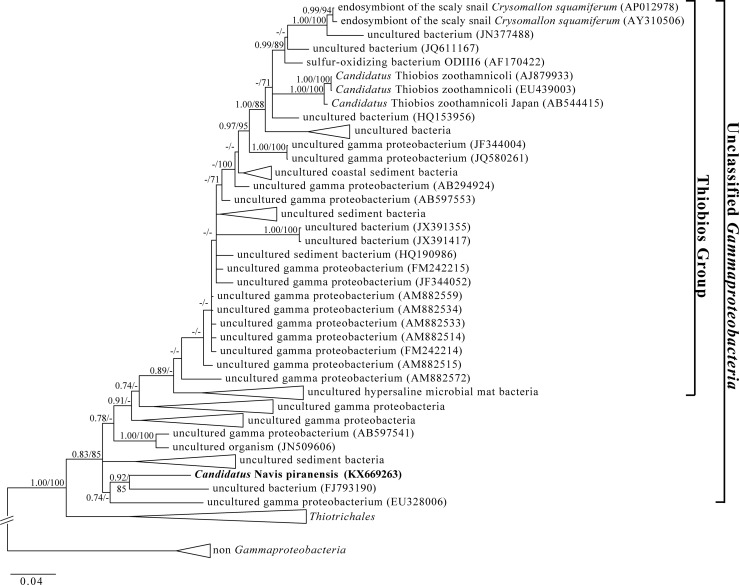
Bayesian inference tree inferred from the nucleotide sequences of the small subunit rRNA gene showing the phylogenetic position of “*Candidatus* Navis piranensis” gen. nov., sp. nov. Numbers at nodes represent Bayesian posterior probabilities and ML bootstrap values. The scale bar corresponds to 4 substitutions per 100 nt positions.

For the symbiont, the obtained 16S rRNA gene sequence had a total length of 1460 nt. Phylogenetic analyses revealed that the ectosymbiont of *Z*. *ignavum* sp. nov. falls into the class of *Gammaproteobacteria*, forming a cluster with two uncultured and unclassified *Gammaproteobacteria* isolated from environmental samples rather than with other ecto- or endosymbionts (Fig 7). Thereby, the closest relative based on sequence similarity is an uncultured bacterium isolated from the Tao Dam hot spring in Thailand (92.1% sequence identity; accession number: FJ793190). Based on this 16S rRNA gene sequence similarity, the results clearly indicated that “*Ca*. Navis piranensis” gen. nov., sp. nov. represents a novel genus and species within a group of unclassified *Gammaproteobacteria*.

### Fluorescence *in situ* hybridization (FISH)

**Fig 8:**
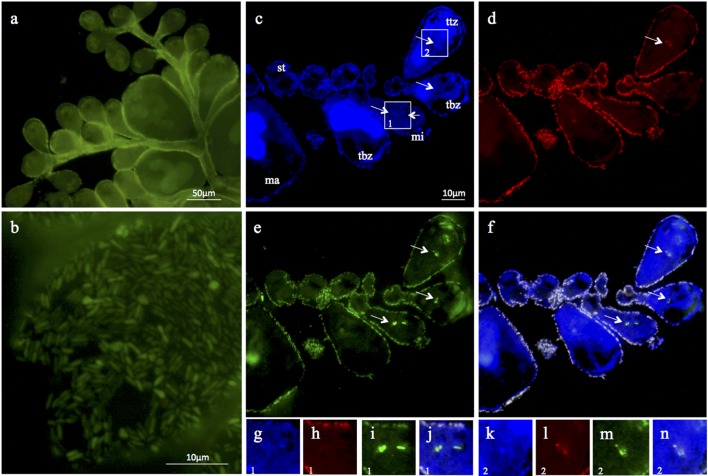
Epi-fluorescent micrographs of the *Zoothamnium ignavum* sp. nov. symbiosis. a, b) LIVE/DEAD staining, c, g, k) DAPI (blue), d, h, l) probe ZIS645 in Cy3 (red), e, i, m) EUBmix in Cy5 (green), f, j, n) overlay with differential interference contrast. ma: macrozooid, mi: microzooid, ttz: top terminal zooid, tbz: terminal branch zooid, st: stalk; the white arrows indicate signals observed in food vacuoles (amplified in g-n).

FISH with both newly designed oligonucleotide probes ZIS645 and ZIS832 confirmed that the obtained sequence originated from the ectosymbiont of *Zoothamnium ignavum* sp. nov. The optimal formamide concentration in the hybridization buffer was found to be 20% for both specific probes. FISH signals from the ectosymbiont-specific probes and the general Bacteria probe set (EUBmix) were similar, indicating that no additional bacteria were present in the bacterial coat on the surface of the colonies, except for the most proximal parts of the stalk. These are apparently overgrown by various unspecific prokaryotes (data not shown). The specific ectosymbionts were found on the stalk, branches, terminal zooids as well as on the macro- and microzooids (Fig 8). The application of probe NON338 (complementary to bacterial probe EUB338) as a negative control yielded no detectable fluorescence signal (data not shown), demonstrating that the signals were not caused by autofluorescence or unspecific staining of the bacteria but rather by specific binding of the probes. Furthermore, FISH signals from the general EUBmix probe set and the ectosymbiont-specific probes were detected within the food vacuoles in several terminal zooids and microzooids (Fig 8). This indicates that *Z*. *ignavum* sp. nov. feeds on both free-living bacteria in the water column and the ectosymbiont.

### Scanning electron microscopy (SEM)

**Fig 9:**
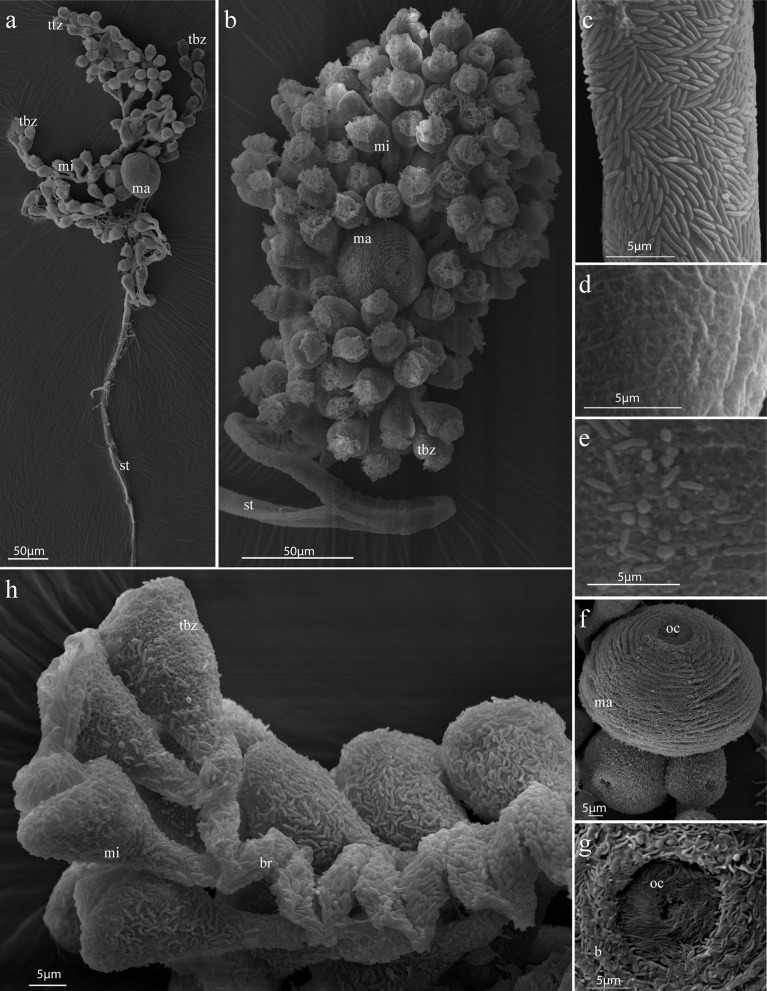
Scanning electron micrographs of *Zoothamnium ignavum* sp. nov. a) Expanded colony, b) contracted colony, c) stalk with bacterial coat, d) aposymbiotic microzooid, e) the two different morphotypes of the ectosymbiont on a microzooid, f) macrozooids, g) oral ciliature of a macrozooid, h) branch with microzooids and the terminal branch zooid. ttz: top terminal zooid, tbz: terminal branch zooid, mi: microzooid, ma: macrozooid, st: stalk, oc: oral ciliature, b: bacteria, br: branch.

The entire colony, except for the most proximal part of the stalk and the adhesive disc was rather fragmentarily covered by symbionts (Fig 9). On the stalk and the branches, the symbiotic coat appeared to consist rather of a bacterial monolayer, while on the micro- and macrozooids the symbionts were mostly found overlapping each other in a multilayer. However, some microzooids also appeared to be completely aposymbiotic. Most cells were rod-shaped bacilli with pointed ends (average length 1.7 μm, SD 0.4 μm; average width 0.4 μm, SD 0.1 μm; n = 520). Occasionally, coccoid-shaped bacteria were found (average diameter 0.6 μm, SD 0.2 μm; n = 70) on the microzooids, especially on the oral side. Rod-shaped bacilli exhibited binary fission at an average length of 2.5 μm (SD 0.4 μm, n = 5), forming two equal daughter cells with an average length of 1.3 μm (SD 0.2 μm, n = 5). Nevertheless, dividing cells were rarely found throughout the colony. Apart from symbiotic bacteria matching in distribution and size to those in FISH sections, an overgrowth of various bacteria could be observed for the lower stalk and the lower branches.

## Discussion

Here, we describe a novel *Zoothamnium* species that was found associated with epibiotic bacteria. The symbiosis was found on sunken wood at two different locations in the North Adriatic Sea (Mediterranean Sea). Collections in spring 2014 and summer 2015 with temperatures between 17.2 and 31.5°C indicate that this symbiosis tolerates very different temperatures. Whether they occur also in fall and especially in winter at lower temperatures needs to be further investigated.

During the second collection in July 2015, *Zoothamnium ignavum* sp. nov. was found on wood pieces co-occurring with *Z*. *niveum* and its thiotrophic gammaproteobacterium “*Ca*. Thiobios zoothamnicoli” [32–37]. While *Z*. *niveum* was found on the strongly degraded parts of the wood where a strong smell of sulfide was noticeable, *Z*. *ignavum* sp. nov. was encountered on less degraded or intact parts of the wood. Occasionally, also free-living white bacteria, most likely sulfide-oxidizing bacteria could be observed (Fig 2).

### The host Zoothamnium ignavum sp. nov.

The genus *Zoothamnium* is characterized by a colonial growth with individual cells connected by a common stalk. Furthermore, the core of the stalk is a continuous spasmoneme, leading to a contraction of the entire colony in a typical ‘zigzag’ pattern [38]. Besides *Zoothamnium*, *Carchesium* Ehrenberg, 1830 is a further colony-developing genus within the peritrich ciliates. However, this genus is characterized by the presence of a discontinuous spasmoneme, leading to a contraction in a helical deformation [55]. In addition to morphological studies, molecular analyses based on the 18S rRNA gene sequence were conducted. Thereby, phylogenetic analyses assigned this novel species to clade II of the family Zoothamniidae (Oligohymenophorea), with the *Z*. *alternans* population from Qingdao (China) being the closest relative (96.7% sequence similarity). Another *Z*. *alternans* population from the USA, however, is rather distantly related to the Qingdao population (96.7% sequence similarity) within clade II [61]. This may suggest that the two *Z*. *alternans* populations represent different species. Therefore, a revision of the current classification of *Z*. *alternans* with detailed morphological comparisons of populations from different geographic locations is necessary.

The polyphyly of *Zoothamnium* was revealed during various molecular DNA analyses of the small subunit of the ribosomal RNA and the internal transcribed spacer (ITS) regions ITS1 and ITS2 [54–57,59–61] and could be confirmed also in this study. Within the five different clades of *Zoothamnium*, species belonging to clade II comprise various symbiotic associations with bacteria: *Z*. *niveum* with the thiotrophic bacterium “*Ca*. Thiobios zoothamnicoli” [32–37], *Z*. *pelagicum* with unknown bacteria assumed to be cyanobacteria [40,88,89,91], and *Z*. *ignavum* sp. nov. with “*Ca*. Navis piranensis” gen. nov., sp. nov. (this study). For *Z*. *alternans* it is unclear whether it is associated with bacteria or not [41,44].

Various kinds of unspecific epigrowth have been observed in other representatives of this genus [46–48], including species from sulfidic marine environments, e.g. *Z*. *entzi* Stiller, 1946, *Z*. *thiophilum* Stiller, 1946, *Z*. *perlatum* Stiller, 1946, and *Z*. *urceolatum* Stiller, 1946, being partially or fully covered by the sulfur-oxidizing bacterium *Thiothrix* Winogradsky, 1888 [49,50]. Consequently, *Zoothamnium* seems to exhibit some kind of tolerance to epigrowth. Also, the nematode family Desmodoridae Filipjev, 1922 is known for microbial fouling [92]. Within this family, all members of the subfamily Stilbonematinae Chitwood, 1936 live in association with specific ectosymbiotic bacteria (reviewed in [92]). The tolerance of epigrowth was suggested to be a prerequisite for the evolution of ectosymbiotic relationships [36,92].

### The ectosymbiont “*Ca*. Navis piranensis” gen. nov., sp. nov.

The 16S rRNA gene sequence analyses and microscopic studies presented in this work revealed that the symbiosis of *Z*. *ignavum* sp. nov. involves a single ectosymbiont species. The colony is rather fragmentarily covered by the symbiont, with certain zooids being fully covered by a monolayer or even a multilayer of symbionts, but others, particularly most recently formed cells on the distal end of the colonies being completely aposymbiotic. In the closely related peritrich *Z*. *niveum*, the ectosymbiont forms a strict monolayer, covering the whole colony except for the most basal part of the colony [33]. In order to sustain a strict monolayer, host growth and symbiont population density must be finely coordinated, in order to prevent overgrowth by or loss of the symbiont [39]. In *Z*. *ignavum* sp. nov. the bacterial layer is highly variable, indicating that the growth of host and symbiont are not well coordinated in this symbiosis.

We observed two different morphologies of the ectosymbiont, rods with pointed ends and cocci with SEM. Coccoid shaped symbionts were especially found on the oral side of the microzooids. Morphological polymorphism is found widespread in symbiotic bacteria, e.g., the thiotrophic endosymbiont of the tubeworm *Riftia pachyptila* (Polychaeta), which is rod-shaped but changes by terminal differentiation into larger cocci, showing transitional stages between the two morphotypes [93,94]. Similarly, in *Z*. *niveum* coccoid rods are restricted to the oral side of the host’s microzooids and rods are found on all other parts of the host [33]. These cell form modulations are considered to be related to nutrition [95]. In the case of thiotrophic symbionts, nutrition means sulfide, oxygen and carbon dioxide for sulfide oxidation and carbon fixation. Differences in nutrition supply might also explain the different morphotypes of “*Ca*. Navis piranensis” gen. nov., sp. nov.

The newly identified *Z*. *ignavum* sp. nov. ectosymbiont forms a monophyletic group with uncultured bacteria isolated from environmental samples rather than with other symbionts. BLASTn analysis revealed that a free-living bacterium isolated from the Tao Dam hot spring in Thailand is the closest relative of “*Ca*. Navis piranensis” gen. nov., sp. nov. (92.1% sequence identity). Other free-living bacteria included in the phylogenetic tree represent uncultured bacteria from different shallow water to deep-sea habitats (Fig 7). Symbiotic bacteria include the endosymbiont of the scaly snail *Crysomallon squamiferum* Chen *et al*., 2015 (Gastropoda) from deep-sea hydrothermal vents in the Indian Ocean [96,97] and the sulfide-oxidizing ectosymbiont of *Zoothamnium niveum* [32–37]. *Zoothamnium niveum* has a cosmopolitan distribution and was reported on or near decaying organic material in tropical to temperate waters [34,36,37,55,98–100]. Whole genome analysis of the scaly snail *Crysomallon squamiferum* endosymbiont revealed the variety of its metabolic capabilities, including hydrogen oxidation and assimilatory ammonification next to sulfur-compound oxidation. Further, pseudogenized genes in the endosymbiont genome included putative ABC transporters of organic compounds and putative sugar phosphotransferase transport systems, suggesting that the symbiont had an ability to grow heterotrophically until recently [101]. The metabolic capabilities of “*Ca*. Navis piranensis” gen. nov., sp. nov. remain to be further studied.

## Supporting Information

S1 Fig Comparison of morphological characteristics of *Z*. *ignavum* sp. nov. with *Z*. *alternans* Qingdao (China) and *Z*. *niveum*. a–c) colony, d–f) microzooid, g–i) oral ciliature. (1): stalk with branches and spasmoneme, (2): stalk without branches and with spasmoneme, (3): stalk without branches and spasmoneme, pm: paroral membrane (haplokinety), am1-3: adoral membranelles 1–3 (polykineties), in: infundibulum, man: macronucleus, s: stomatogenic kinety. b, e: modified from [36]; h: reused from [82]; c, f, i: modified from [44].
(TIF)

S1 Table Accession numbers of the sequences included in the 18S rRNA gene sequence phylogenetic analysis. (DOCX)

S2 Table Accession numbers of the sequences included in the 16S rRNA gene sequence phylogenetic analysis. (DOCX)
