# Correction: A Novel Colonial Ciliate *Zoothamnium ignavum* sp. nov. (Ciliophora, Oligohymenophorea) and Its Ectosymbiont *Candidatus* Navis piranensis gen. nov., sp. nov. from Shallow-Water Wood Falls

**DOI:** 10.1371/journal.pone.0167873

**Published:** 2016-12-01

**Authors:** The PLOS ONE Staff

There are errors in the Subclass and Order information presented in the ciliate classification table in the Results section. Please see the corrected ciliate classification table here. The publisher apologizes for the errors.

The ciliate classification follows Lynn [38].

**Table:** 

Phylum:	Ciliophora Doflein, 1901
Class:	Oligohymenophorea de Puytorac et al., 1974
Subclass:	Peritrichia Stein, 1859
Order:	Sessilida Kahl, 1933
Family:	Zoothamniidae Sommer, 1951
Genus:	*Zoothamnium* Bory de Saint-Vincent, 1824
