# Supplementary material for: A Novel Colonial Ciliate Zoothamnium ignavum sp. nov. (Ciliophora, Oligohymenophorea) and Its Ectosymbiont Candidatus Navis piranensis gen. nov., sp. nov. from Shallow-Water Wood Falls
Source: PLoS One. 2016 Sep 28;11(9):e0162834. doi: 10.1371/journal.pone.0162834 (PMC5040259; doi:10.1371/journal.pone.0162834)
Supplement: S1 Table — (DOCX) [file pone.0162834.s002.docx]

| **accession number** | **organsim** |
| --- | --- |
| KX669262 | *Zoothamnium ignavum* |
| KM887956 | *Zoothamnium parahentscheli* |
| KF790904 | *Zoothamnium intermedium* |
| KM222118 | *Zoothamnium hentscheli* |
| KM887954 | *Zoothamnium grossi* |
| KM887955 | *Zoothamnium arcautum* |
| AY049000 | *Astylozoon enriquesi* |
| EF417834 | *Opisthonecta minima* |
| X56531 | *Opisthonecta henneguyi* |
| DQ868347 | *Vorticella microstoma* |
| JN120203 | *Vorticellides infusionum* |
| GQ872427 | *Vorticellides* *astyliformis* |
| JQ723990 | *Vorticellides aquadulcis* |
| GQ872429 | *Vorticella gracilis* |
| DQ190468 | *Vorticella fusca* |
| DQ868348 | *Vorticella convallaria* |
| GU987024 | *Vorticella* sp. 2 PPS-2010 |
| GU187049 | *Vorticella* sp. SP-2009-2 |
| DQ662849 | *Vorticella campanula* |
| DQ845295 | *Pseudovorticella sinensis* |
| DQ662847 | *Pseudovorticella paracratera* |
| DQ190466 | *Pseudovorticella punctata* |
| DQ190462 | *Epicarchesium abrae* |
| AF401526 | *Ophrydium versatile* |
| GQ221940 | *Apocarchesium arndti* |
| GU187056 | *Apocarchesium rosettum* |
| AF401522 | *Carchesium polypinum* |
| AF401523 | *Zoothamnium arbuscula* |
| DQ868350 | *Zoothamnium niveum* |
| DQ662855 | *Zoothamnium alternans* QD pop-2 |
| DQ662850 | *Zoothamnium alternans* QD pop-1 |
| DQ868351 | *Zoothamnium pelagicum* |
| DQ662854 | *Zoothamnium plumula* |
| DQ868352 | *Zoothamnium alternans* USA |
| AF335516 | *Epistylis urceolata* |
| AF335515 | *Epistylis wenrichi* |
| HM627237 | *Epistylis* sp. 1 LRPU-2010 |
| AF335514 | *Epistylis chrysemydis* |
| GQ872428 | *Peritrichia* sp. TS-2009a |
| EF417835 | *Telotrochidium matiense* |
| DQ662853 | *Zoothamnium pararbuscula* |
| DQ868356 | *Zoothamnium* sp. JCC-2006-5 |
| DQ868353 | *Zoothamnium* sp. JCC-2006-1 |
| AY319769 | *Zoothamnopsis sinica* |
| DQ190469 | *Zoothamnopsis sinica* |
| DQ662851 | *Zoothamnium duplicatum* |
| DQ868354 | *Zoothamnium* sp. JCC-2006-2 |
| JN836351 | *Myoschiston duplicatum* |
| JN836352 | *Myschiston cf. duplicatum* |
| DQ662852 | *Zoothamnium* *nii* |
| DQ868355 | *Zoothamnium* sp. JCC-2006-4 |
| AF401521 | *Vaginicola crystallina* |
| HM627241 | *Opercularia* sp. 3 LRPU-2010 |
| HM627240 | *Opercularia* sp. 2 LRPU-2010 |
| HM627239 | *Opercularia* sp. 1 LRPU-2010 |
| HM627238 | *Opercularia allensi* |
| AF401525 | *Opercularia microdiscum* |
| AF401527 | *Epistylis galea* |
| AF401524 | *Campanella umbellaria* |
| JX178767 | *Zoothamnium* sp. 1 JG-2011 |
| KU363270 | *Zoothamnium* sp. 1 PS-2016 |
| JX178768 | *Zoothamnium* sp. 2 JG-2011 |
| KU363271 | *Zoothamnium* sp. 2 PS-2016 |
| KU363274 | *Zoothamnium* sp. 3 PS-2016 |
| KU363273 | *Zoothamnium* sp. 4 PS-2016 |
| JQ956531 | *Zoothamnium* sp. HN Z1 |
| KU363255 | *Zoothamnium* sp. PS-2016a |
