# Supplementary material for: A Novel Colonial Ciliate Zoothamnium ignavum sp. nov. (Ciliophora, Oligohymenophorea) and Its Ectosymbiont Candidatus Navis piranensis gen. nov., sp. nov. from Shallow-Water Wood Falls
Source: PLoS One. 2016 Sep 28;11(9):e0162834. doi: 10.1371/journal.pone.0162834 (PMC5040259; doi:10.1371/journal.pone.0162834)
Supplement: S2 Table — (DOCX) [file pone.0162834.s003.docx]

| **accession number** | **organism** |
| --- | --- |
| KX669263 | “*Ca.* Navis piranensis” |
| AY310506 | endosymbiont of the scaly snail *Crysomallon squamiferum* |
| AP012978 | endosymbiont of the scaly snail *Crysomallon squamiferum* |
| AB597541 | uncultured gamma proteobacterium |
| AB597527 | uncultured gamma proteobacterium |
| FJ793190 | uncultured bacterium |
| HF558546 | uncultured bacterium |
| JQ579995 | uncultured gamma proteobacterium |
| AM882521 | uncultured gamma proteobacterium |
| KR825102 | uncultured bacterium |
| JQ579851 | uncultured gamma proteobacterium |
| JF344548 | uncultured gamma proteobacterium |
| FJ437956 | uncultured bacterium |
| HQ191054 | uncultured sediment bacterium |
| AM882560 | uncultured gamma proteobacterium |
| FJ437979 | uncultured bacterium |
| KT257846 | uncultured bacterium |
| JN509606 | uncultured organism |
| HQ191083 | uncultured sediment bacterium |
| FJ717238 | uncultured bacterium |
| FJ437990 | uncultured bacterium |
| HQ191084 | uncultured sediment bacterium |
| AM882559 | uncultured gamma proteobacterium |
| AF170422 | sulfur-oxidizing bacterium ODIII6 |
| FJ717231 | uncultured bacterium |
| AM882515 | uncultured gamma proteobacterium |
| JN977209 | uncultured bacterium |
| AM882565 | uncultured gamma proteobacterium |
| JQ580036 | uncultured gamma proteobacterium |
| AM882534 | uncultured gamma proteobacterium |
| NR_116699 | *Thiohalobacter thiocyanaticus* |
| EU491264 | uncultured bacterium |
| AM882533 | uncultured gamma proteobacterium |
| JF344004 | uncultured gamma proteobacterium |
| HQ191006 | uncultured sediment bacterium |
| KX097365 | uncultured bacterium |
| JX226822 | uncultured bacterium |
| JQ269275 | bacterium WHC2-2 |
| AM882514 | uncultured gamma proteobacterium |
| FM242214 | uncultured gamma proteobacterium |
| HQ191056 | uncultured sediment bacterium |
| GQ274161 | uncultured gamma proteobacterium |
| KR825155 | uncultured bacterium |
| AB597553 | uncultured gamma proteobacterium |
| JF344461 | uncultured gamma proteobacterium |
| JF344427 | uncultured gamma proteobacterium |
| AM882571 | uncultured gamma proteobacterium |
| HQ191050 | uncultured sediment bacterium |
| HQ190986 | uncultured sediment bacterium |
| FM242215 | uncultured gamma proteobacterium |
| KR825164 | uncultured bacterium |
| JQ579815 | uncultured gamma proteobacterium |
| JF344496 | uncultured gamma proteobacterium |
| EU328006 | uncultured gamma proteobacterium |
| AB294924 | uncultured gamma proteobacterium |
| HE804020 | uncultured gamma proteobacterium |
| JF344361 | uncultured gamma proteobacterium |
| JF344128 | uncultured gamma proteobacterium |
| HQ191010 | uncultured sediment bacterium |
| HQ190999 | uncultured sediment bacterium |
| FJ545460 | uncultured bacterium |
| AM882572 | uncultured gamma proteobacterium |
| KT880265 | uncultured gamma proteobacterium |
| KC238391 | uncultured bacterium |
| JQ013161 | uncultured bacterium |
| JF344213 | uncultured gamma proteobacterium |
| JF344187 | uncultured gamma proteobacterium |
| AM040135 | uncultured gamma proteobacterium |
| HE803932 | uncultured gamma proteobacterium |
| JF344296 | uncultured gamma proteobacterium |
| JN977193 | uncultured bacterium |
| KT880462 | uncultured bacterium |
| GQ274309 | uncultured gamma proteobacterium |
| FM211756 | uncultured gamma proteobacterium |
| EU491126 | uncultured bacterium |
| JF344065 | uncultured gamma proteobacterium |
| GQ249522 | uncultured gamma proteobacterium |
| KR825100 | uncultured bacterium |
| HM598246 | uncultured bacterium |
| KT880456 | uncultured bacterium |
| JF344052 | uncultured gamma proteobacterium |
| EU491205 | uncultured bacterium |
| HQ703856 | uncultured bacterium |
| JN471637 | uncultured organism |
| AJ879933 | “*Candidatus* Thiobios zoothamnicoli” |
| EU439003 | “*Candidatus* Thiobios zoothamnicoli” |
| AB544415 | “*Candidatus* Thiobios zoothamnicoli” Japan |
| JN377488 | uncultured bacterium |
| JQ611167 | uncultured bacterium |
| HQ153956 | uncultured bacterium |
| JQ269282 | bacterium WHC3-6 |
| JQ580261 | uncultured gamma proteobacterium |
| JQ579858 | uncultured gamma proteobacterium |
| HQ191022 | uncultured sediment bacterium |
| JX391355 | uncultured bacterium |
| JX391417 | uncultured bacterium |
| JN427747 | uncultured organism |
| JN447965 | uncultured organism |
| GQ354996 | uncultured Thiotrichales bacterium (Thiotrichales) |
| FR847876 | “*Candidatus* Thiopilula sp. ST-S116” (Thiotrichales) |
| FN811660 | “*Candidatus* Thiopilula aggregata” (Thiotrichales) |
| EU919200 | “*Candidatus* Allobeggiatoa halophila” (Thiotrichales) |
| NR_025830 | *Rhodospirillum centenum* (OUTGROUP) |
| AB015420 | *Sinorhizobium indiaense* (OUTGROUP) |
| DQ403194 | *Sinorhizobium meliloti* (OUTGROUP) |
| D30778 | *Rhodospirillum rubrum* ATCC 11170 (OUTGROUP) |
